# Multiomics Insights in Research of Nitric Oxide (NO) Donors in Plants upon Stress

**DOI:** 10.3390/ijms27198897

**Published:** 2026-10-07

**Authors:** Anastasia Gurina, Anna Margarit, Anastasia Zhikhreva, Nadezhda Frolova, Andrej Frolov

**Affiliations:** 1 Federal State Institution Federal Research Centre “Fundamentals of Biotechnology”, Russian Academy of Sciences, 119071 Moscow, Russia; ancka.margarit@yandex.ru (A.M.); anastasiazhikhreva@gmail.com (A.Z.); 2 Laboratory of Analytical Biochemistry and Biotechnology, K.A. Timiryazev Institute of Plant Physiology Russian Academy of Science, 127276 Moscow, Russia; frolovanadja@yandex.ru; 3 Institute of Life Sciences, Immanuel Kant Baltic Federal University, 236041 Kaliningrad, Russia

**Keywords:** multiomics, nitric oxide, NO donors, *S*-nitrosylation, phytoeffectors

## Abstract

It is known that the progressive climate changes and environmental pollution prevalent all over the world lead to increasingly frequent and prolonged episodes of stress, and it challenges plants to adapt to changing adverse conditions. Given the significant crop losses caused by challenging environments, protecting plants from the destructive effects of these factors has become one of the most pressing challenges in modern agricultural biology and plant physiology. One of the approaches to protect plants from stressors in modern agrobiotechnology is the use of nitric oxide (NO) donors, which are chemicals that can release NO in plant cells and stimulate their germination, growth, biomass accumulation, and protection against external factors. Studying their mechanism of action at the molecular level is essential for understanding the targets of NO action, as well as for developing new phytoeffectors. A multiomics approach, along with physiological data, is often the method of choice. In this review, we compare widely used NO donors (e.g., sodium nitroprusside, *S*-nitrosoglutathione, NONOates) in terms of chemical properties, NO-release kinetics, stability, and suitability for different application methods (seed treatment, foliar spray, root application). Finally, we highlight recent advances from proteomics, metabolomics, and transcriptomics that have enabled system-wide identification of NO-responsive pathways and candidate targets. By integrating physiological, biochemical, and multiomics data, this review provides a framework for understanding NO-mediated stress tolerance and for the rational design of new NO-based phytoeffectors.

## 1. Introduction

During their life cycle, plants are constantly exposed to negative environmental factors called stressors. These can include a variety of abiotic factors (drought, flooding, high salt levels, heavy metals, and heat) and biotic factors (predators, fungi, and bacteria). Due to their sessile nature, plants have developed numerous adaptations to cope with these challenging environmental factors. Among them are various enzymatic and non-enzymatic antioxidant defense systems [1]. The principal antioxidant enzymes include catalase (CAT), superoxide dismutase (SOD), ascorbate peroxidase (APX), glutathione peroxidase (GPX), glutathione reductase (GR), glutathione *S*-transferases (GST), monodehydroascorbate reductase (MDHAR), and dehydroascorbate reductase (DHAR) [2]. Non-enzymatics defense systems include specialized secondary metabolites (e.g., glutathione, carotenoids, ascorbic acid, tocopherols, and flavonoids) [3]. Nevertheless, plants frequently struggle to withstand these pressures as a result of escalating climate shifts, industrial contamination, shifts in nutrient levels, and pest outbreaks. This is especially critical for food crops, where the inability of plants to combat stress leads to a loss of productivity, reduced crop quality, or even complete crop loss. 

Protecting plants from the destructive effects of these factors is one of the most pressing challenges in modern agricultural biology and plant physiology. There are two approaches that can be used to address this issue. On the one hand, modern breeding techniques have led to the development of new varieties that are highly resistant to stressful conditions. On the other hand, the agrobiotechnological approach continues to explore the concept of the so-called phytoeffector approach, which involves the use of various chemical stress response modulators, including synthetic ones. At the molecular level, the basis of their action may lie in the activation of specific signaling and protective cascades, as well as the direct modulation and/or inhibition of specific enzymes, which, in turn, can significantly reduce the harmful effects of stress. Thus, the spectrum of possible biochemical mechanisms of action of phytoeffectors is largely similar to that of pharmaceutical drugs used in medicine. However, given the complexity of plant metabolism and regulatory systems, an adequate and comprehensive understanding of the protective properties of specific substances requires evaluation of their effects at the physiological and biochemical levels.

One of the groups of phytoeffectors are substances that can release nitric oxide (NO) when applied exogenously. NO is a signaling molecule in plant cells, and it is currently known that NO donors can modulate the activity of antioxidant enzymes (superoxide dismutase (SOD), catalase (CAT), peroxidase (POD), ascorbate peroxidase), decrease lipid peroxidation, induce osmolyte accumulation, regulate stomata closure, and maintain photosynthetic efficiency [4,5]. Moreover, nitric oxide acts through protein modifications (mainly, *S*-nitrosylation), which influence protein function, stability, localization and activity [6], and multiple targets were identified to date. Studying their functional role has revealed that *S*-nitrosylation regulates the synthesis and signaling of phytohormones, immunity, programmed cell death, RNA splicing, and gene expression [7,8,9,10]. Thus, now a functional versatility of this small molecule suggests that it exists in a complex internal network of interactions between hormones, protein signaling cascades, and transcription factors (TFs). 

Research on the role of nitric oxide in protecting plants from stressors would not have been possible without the use of exogenous donors. The most widely used donors include sodium nitroprusside (SNP), *S*-nitroso-*N*-acetyl-*D,L*-penicillamine (SNAP), and *S*-nitrosoglutathione (GSNO) [11]. Despite their proven efficiency *in planta*, their further employment in nitric oxide research was called into question multiple times. For example, SNP releases cyanide and iron besides NO and thereby could not be considered as a “clear” NO donor, and the action could not be attributed surely to NO [12]. In this regard, these and other shortcomings of existing phytoeffectors have led scientists to search for new formulas or methods of delivering substances to plant tissues. For example, relatively recently, the synthesis of compounds from the diazeniumdiolates (NONOates) and sydnoneimines groups has been highlighted in the literature, and the first studies on plants proved their beneficial effects [13,14]. However, these studies are still in the early stages, and further research is needed to confirm their effectiveness.

Rapid screening of many newly synthesized substances requires simple, relatively inexpensive, and preferably non-invasive methods. For plants, those include seed germination, root elongation, and early seedling growth assays [15,16], while the preferred non-invasive approaches are red–green–blue imaging (RGB), chlorophyll fluorescence, and hyperspectral imaging for repeated measurements on living plants [17,18]. Although performative as a first assessment procedure, it does not provide any mechanistic insight. From this perspective, a multiomics approach is often the best way to solve this problem. Multiomics approaches provide a comprehensive view of genetic information flow, enabling researchers to evaluate how specific nitric oxide donors influence gene expression patterns (transcriptomics). They also could reveal protein abundance changes and post-translational modification of proteins (proteomics) [19]. Although metabolomics does not directly involve “reading” genetic information, it studies the set of low-molecular-weight metabolites (end products and intermediate metabolic products) in a cell—precisely the final biochemical “fingerprint” that remains after the entire regulatory system has operated: genes, proteins, and external signals [20]. This review aims to highlight the available data on NO signaling and known molecular targets, summarize the practical application of known NO donors, and report on new achievements in this field, as well as to showcase the existing research on NO signaling using a multiomics approach.

## 2. Endogenous Nitric Oxide (NO) in Plant Signaling Systems

The physiological importance of nitric oxide in plants has been debated for years, despite its initial discovery in animal systems [21]. However, as techniques for detecting free radicals in cells have evolved and experimental data have accumulated, it has become clear that NO also plays a significant role in various plant processes, from seed germination to defense mechanisms in response to stress [22]. These advancements have led to the study of NO signaling as a vibrant field in biochemistry and physiology. The development of sensitive detection methods, such as electron paramagnetic resonance (EPR) spectroscopy and diaminofluorescein (DAF)-based fluorescent probes, has allowed researchers to experimentally confirm the production of NO in plant tissues [23,24]. 

As in animals, nitric oxide can be synthesized in plants through various pathways, including both enzymatic and non-enzymatic mechanisms. Recent advances in this field strongly indicate that the NO signaling network is much more complicated than the simple “NO-to-cGMP” model, and a shift toward a compartmentalized redox signaling framework in which *S*-nitrosylation, GSNO turnover, denitrosylation, ERFVII/PRT6-based (ethylene response factor group VII/PROTEOLYSIS 6) sensing, and chromatin regulation carry much of the signal is evident [25,26]. Nitrite reduction is one of the main pathways for NO synthesis in land plants. Non-enzymatic reduction of nitrite occurs under low-pH conditions or in highly reducing environments with high concentrations of nitrite, and while these conditions are rarely encountered in living organisms, the amounts of NO produced through this mechanism can be significant (e.g., in aleurone layers of seeds) [27]. The enzymatic reduction of nitrate is carried out by nitrate reductase (NR), which is thought to be the main source of nitric oxide signal evolution in photosynthetic organisms. NR is a multifunctional enzyme involved in nitrogen assimilation and metabolism, and it catalyzes the reduction of nitrate to nitrite using NADH as an electron donor [28]. NR also has nitrite reductase activity, catalyzing the reduction of nitrite to NO, although this reaction is more of a side process under normal conditions [29]. However, under hypoxic conditions, NR activity significantly contributes to NO production [29]. Studies on *Arabidopsis thaliana* mutants with impaired NO production have confirmed these findings. For example, *nia1*/*nia2* mutants (with impaired NR function) exhibit impaired NO-mediated stress responses [30,31]. In addition, a complex between NR and NOFNiR (NO-forming nitrite reductase) has been discovered in *Chlamydomonas reinhardtii*, which performs the reduction of nitrite to NO [32]. Another pathway for the production of NO, the oxidative pathway, involves the use of arginine and is similar to the main pathway for the synthesis of NO in animals using NO-synthase (NOS). However, the presence of NOS-like activity in plants is a subject of debate [33]. Despite reports of NOS-like activity, no genes or proteins with homology to animal NOS have been identified in higher plants to date [28]. *AtNOS1* was previously described as potential nitric oxide synthase in *Arabidopsis thaliana* but was proved to be GTPase, which is supported by the studies with orthologous *YqeH* protein from *Geobacillus* [34]. Recent studies have identified NOS-like proteins in algae, such as *Ostreococcus tauri* and other *Mamiellophyceae* [35], *Synechococcus* sp. [36], suggesting that plants may have unique NOS that differ from those found in animals [37]. Some studies indicate that NOS-like activity in plants could be observed due to other enzymes, such as xanthine oxidoreductase, peroxidases, cytochrome P450, and some heme-containing proteins [38].

Nitrite reduction also occurs enzymatically in electron transport chains (ETCs) of chloroplasts and mitochondria [39]. For example, mitochondria can generate NO in the ETC under anaerobic conditions, primarily through complexes III and IV [40]. Non-enzymatic NO conversion from nitrite occurs in the presence of cellular reductants such as ascorbate, so the chemistry of an organism itself can become a signal source under favorable conditions [5]. To date, plant NO is reported to be generated in chloroplasts, mitochondria, peroxisomes, cytoplasm, cell wall, plasma membrane, vacuoles, apoplasts, and diverse internal membranes, and this compartmentalization could be an additional level of the general signaling system [41].

## 3. Molecular Bases of Nitric Oxide Signaling in Plant Cells

Nitric oxide acts as a signaling molecule, triggering various signal transduction pathways in cells. These pathways can be divided into two main groups: those dependent on the secondary messenger cyclic guanosine monophosphate (cGMP) and those independent of it [42]. Other NO-dependent signal transduction routes include metal nitrosylation, tyrosine nitration, formation of nitro-fatty acids, peroxynitrite formation, and nitrated nucleotides such as 8-nitro-cGMP [25,43]. Additionally, a conserved animal guanylate cyclase pathway was still not confirmed in plants and seems not to play any significant role in NO signaling in plants [25].

Due to the ability to move between cells and interact with protein targets, NO plays a crucial role in many plant processes. These include growth and development, responses to stress, and diverse metabolic pathways [44]. The signaling role of nitric oxide in plant cells is currently evident, and it performs this role primarily through the post-translational modifications (PTMs) of proteins, the main part of which is *S*-nitrosylation [45]. This means that *S*-nitrosylation has a direct effect on protein functions, their interactions, and localization [46]. On a larger scale, it affects the functioning of different protein regulators in various signaling pathways, as was shown for phytohormones, stress responses, immune reactions, and many other crucial plant processes [7]. Nitrosylated glutathione (GSNO) acts as the main NO carrier, as well as its reservoir [47]. GSNO is the main low-molecular-weight thiol in plant cells and is found in various organelles, including the cytosol, chloroplasts, mitochondria, and peroxisomes [23,48]. GSNO is a highly mobile NO signal transporter between plant cells and organs, which can be formed in response to various stressors [47]. Phytoglobins (Pgbs) are involved in the metabolic cycle of nitric oxide conversion, in which the enzyme nitrate reductase plays a key role. This cycle, known as the Pgb–NO cycle or Pgb–NO respiration, helps plants maintain their energy status under conditions of limited oxygen access (hypoxia or anoxia) [49]. NR converts nitrate to nitrite, which is transported to the mitochondria, where it replaces oxygen as the final electron acceptor, resulting in the reduction of nitrite to NO [50].

### 3.1. Nitric Oxide as a Mediator of Stress Responses

At the moment, it is clear that nitric oxide is a signaling molecule that regulates various events not only in normal conditions, but also under stress. It is involved in hormonal signaling crosstalk, transcriptional control of some target genes, interplay with reactive oxygen species (ROS) signaling, and regulation of activity of proteins through *S*-nitrosylation [51]. Once a stressful event occurs, NO rises endogenously, but an outcome for the particular case depends on concentration, timing, enzymatic control, and cellular redox capacity [52]. Nevertheless, depending on these factors, nitric oxide could provide acclimation under unfavorable conditions [52].

Interactions of ROS and NO give rise to various reactive nitrogen species (RNS) [53]. Both ROS and RNS are divided into radical and non-radical forms. Superoxide anion, hydroxyl, peroxyl and hydroperoxyl are the most common ROS radicals, whereas hydrogen peroxide, hypochlorous acid, singlet oxygen, and hypoperoxide are non-radical representatives. From RNS, radicals include nitric oxide, nitrate, and nitric dioxide, while peroxynitrate, nitrous acid, nitroxyl anion, and dinitrogen tetroxide are non-radicals [54]. ROS and RNS are commonly accumulated after a stress onset, which enables their orchestrated regulation of stress responses [55]. One of the principal events in ROS–RNS interconnection is a rapid reaction of NO with superoxide anion radical, generating peroxynitrite—strong oxidant and nitrating agent [56]. Peroxynitrite formation is a source of tyrosine nitration in proteins and nitrosative stress (although it could have regulative effects at basal levels) [55]. Moreover, *S*-nitrosylation events modulate ROS production directly, for example, as shown for NADPH oxidase, which controls cell death in plant immunity [54]. Collectively, through the formation of GSNO, *S*-nitrosylation, and protein tyrosine nitration, ROS–RNS interactions can modify the activity of redox-regulated proteins and thereby influence cellular signaling and stress-related damage [55]. 

One of the earliest findings was an involvement of NO in abscisic acid (ABA)-dependent stomata closure under drought conditions. ABA positively regulates NO production, and the latter contributes to reduce transpirational water loss alongside calcium signaling, ROS, and mitogen-activated protein kinases (MAP kinases) [57,58]. More recent findings indicate that *CLE9*-dependent stomata closure is also dependent on NO as a messenger [59]. Exogenous application of the peptide failed to induce NO levels and closure of stomata in NR mutants, showing the direct involvement of CLE9 peptide in the induction of endogenous NO formation. Beyond stomata regulation, NO contributes to the improvement of antioxidant capacity, osmolyte accumulation, regulation of root architecture, and involved induction of ABA-dependent drought tolerance genes such as *Arabidopsis thaliana* aldehyde oxidase *AtAO3* [51,53,60]. ABA-driven drought signaling is negatively regulated by endogenous NO through *S*-nitrosylation of the core kinase OST1/SnRK2.6, which inhibits ABA-induced stomatal closure. Accordingly, *gsnor* mutants accumulate excess NO, show impaired stomatal closure, and lose more water under stress [61]. At the same time, some reviews emphasize that NO might not be critical in rapid stomata closure [53]. These observations might be another piece of the puzzle in a complicated signaling network where outcomes of NO upbuilding are very context-dependent. 

In addition to providing drought protection, NO action aims to combat oxidative damage. The comprehensive meta-analysis of 86 studies addressing heavy metal stress highlights that exogenous NO supplementation increased biomass and stimulated antioxidant enzymes. It also raised Ca, Fe, and Mg contents and reduced heavy metal accumulation in plant tissues [62]. Osmotic stress (e.g., salinity) models follow the same patterns: NO modulates ion homeostasis, antioxidant defense, and stress-relative gene expression involving crosstalk with phytohormones [51]. In tomato seedlings exposed to high salinity, GSNO treatment improved tolerance, which was explained by protein *S*-nitrosylation and transcriptional reprogramming of MAPKs and carbohydrate metabolism [63].

Furthermore, NO affects the oscillators (oscillating elements) of circadian rhythms. Although it is not part of the molecular “core” of circadian oscillators, NO acts more as a modulator and intermediary of external signals, indirectly but noticeably altering the functioning of the biological clock. In particular, it reduces the amplitude of signals from the GI (GIGANTEA) and CO (CONSTANS) genes, which are precisely the key components of these clocks. As a result, the coordination between the plant’s internal biological clocks and external conditions (light, temperature) is disrupted, which may ultimately lead to a delay in flowering [64].

### 3.2. Role of Protein *S*-Nitrosylation in the Regulation of Plant Stress Responses

The study of *S*-nitrosylation of proteins is a separate field of research of NO signaling, which provides a specific answer to the question of how the differential modification of proteins is related to certain effects on plants. Existing research on *Arabidopsis thaliana* is notably more robust, offering comprehensive *S*-nitrosylation profiles across a wide range of stress conditions [65,66,67]. One of the earliest studies, utilizing GSNO application as a NO source, demonstrated 63 and 52 candidate nitrosylated proteins from cell culture and leaf tissue, respectively, which were attributed to specific cellular functions including stress- and redox-related, signaling, cytoskeleton regulation, and metabolic interconversions [67]. A later study employed GSNO reductase 1 (*GSNOR1*) *A. thaliana* mutants and identified more than a thousand *S*-nitrosylated peptides matching 926 proteins in leaves and showed that they were involved in chlorophyll metabolism, photosynthesis, carbohydrate metabolism, and stress responses [68]. 

While full-scale proteomics studies provide a general overview of all possible *S*-nitrosylation spots, functional studies address particular effects of the modification. It was found that many nitrosylation spots lie in antioxidant and redox systems, supporting the role of this modification in fine-tuning ROS and RNS balance during stress. The case study on ascorbate peroxidase 1 (AtAPX1) using *gsnor1-3* mutants (accumulating excessive endogenous NO) showed that modification of Cys32 of this protein with NO enhanced its activity and improved antioxidant defense during stress [69]. 

*S*-nitrosylation could target plant immunity and transcriptional factors: the *Arabidopsis* nonexpressor of pathogenesis-related genes 1–transcription factor TGA1 (NRP1–TGA1) system of immunity response was *S*-nitrosylated after GSNO application, but in different ways. NRP1 translocation to the nucleus was promoted by NO, whereas *S*-nitrosylation on Cys260/Cys266 protected TGA1 from oxidative damage and increased its DNA-binding capacity [70]. *S*-nitrosothiol regulated gene 1 (SRG1) is another protein of the immunity system of *Arabidopsis*: persistent endogenous NO (upon pathogen attack or in *gsnor* mutants) drives nitrosylation at Cys87, which releases SRG1 DNA-binding capacity and transcriptional repression [71]. Those two examples in the context of plant immunity clearly indicate that NO modifications of proteins are not simple “turn on/off” switches of their activity but also regulators of binding, transport, and transcriptional activity. 

Core components of nitrate sensing and assimilation are subject to NO-dependent regulation. Nitrate metabolism is of particular interest as the main components of this system are regulated by *S*-nitrosylation in *Arabidopsis* and are highly responsive to stress [72]. NIN-LIKE PROTEIN 7 (NLP7), the primary nitrate response master regulator, is modified with NO in vitro in *Arabidopsis*, and NO is required for expression of other genes in this pathway—NADH nitrate reductase 1 (NIA1), NO-forming nitrite reductase (NIR), nitrate transporter 1.1 (NRT1.1), and NRT2.1 (nitrate transporter 2.1) [73]. Moreover, NO, which is formed as a product during the operation of nitrate reducatse, can *S*-nitrosylate this enzyme itself. Modification of cysteine residues in the structure of nitrogenase leads to its inactivation [74]. That is, the cell thus “senses” the level of NO and, in the event of its excess, “dials down” the activity of the enzyme that produced it. This example places NO into a larger metabolic and autoregulation pathway. 

Regulation of functions by *S*-nitrosylation also affects individual metabolic pathways. Studies of monosaccharide catabolism have revealed that NO indeed suppresses the breakdown of monosaccharides. The mechanism lies precisely in *S*-nitrosylation, which was shown both with NO donors and NO-overproducing mutants: NO modifies enzymes that are involved in sugar metabolism (for example, key enzymes of glycolysis or starch synthesis pathways), thereby slowing down their activity. As a result, starch may accumulate in the cell—this is one of the ways in which the plant regulates carbohydrate metabolism in response to NO signals [75,76]. In general, *S*-nitrosylation is a flexible tool to finely regulate a wide variety of processes—from energy metabolism to developmental rhythms and nitrogen metabolism.

## 4. Exogenous Nitric Oxide Donors: Types, Chemistry, and Functional Comparison

### 4.1. Physicochemical Properties of the Main Nitric Oxide Donors

As it could be seen from the examples in this review, most of the studies employ exogenous donor application to study NO protective effects and *S*-nitrosylation patterns. That is, different NO donors are a valuable tool in nitric oxide research. Overall, the main characteristics of the most usable NO donors are summarized in Table 1.

The most widely employed plant NO donors can be divided into inorganic nitrosyl donors [99], *S*-nitrosothiols [100], diazeniumdiolates/NONOates [101], and controlled release systems [102], while organic nitrate/nitrite donors are less used in plant experimental practice and are usually discussed in the context of endogenous nitrite-dependent NO formation [103]. This variety of nitric oxide donors is due to the fact that they all exhibit different NO release kinetics and stability, also dependent on different physicochemical factors (such as light and temperature) [78], and undesired adverse effects [104]. 

Apparently, all the NO donors exhibit dose-dependent effects on plants [83]. They have a particular threshold of concentrations above which ruinous consequences outweigh beneficial impact [84]. These outcomes result mainly from nitrosative stress. It leads to growth retardation [85], lipid peroxidation [82], changes in activity of antioxidant enzymes [87], and cell death [88]. The concentration range of NO donors utilized in plant experiments is broad, typically varying from micro- to millimolar levels due to differences in chemical structure, exposure time, pH, light, plant species, and delivery means. SNP is often used at micromolar up to several millimoles, whereas GSNO is usually applied at lower concentrations than SNP because it ensures a slower and more sustained NO signal and is frequently used in studies of redox regulation and protein *S*-nitrosylation [89]. Effective doses of NONOates are strongly defined by the timing of NO liberation determined by certain derivative [86], which makes them especially useful for kinetic and mechanistic experiments. That is, the choice of donor and its concentration should be carefully selected for a specific study. 

Sodium nitroprusside (SNP) is the most widely used NO donor in plant research [97,98]. It is reported that SNP has relatively high half-life periods in solution (up to 12h) and high photosensitivity [91]. Some studies also indicate that SNP’s NO release in biological systems is dependent on sulfhydryl-containing substances such as GSH or cysteine residues of proteins [92,93]. It is a well-known fact that SNP releases cyanide and iron alongside NO [95]. Cyanide release could be followed by a reduction in photosynthetic capacity [95] and other possible negative outcomes associated with overaccumulation of this ion. However, cyanide has not only detrimental effects on plants, but it also interrupts the dormancy period of seeds [96]. Although the effects of SNP-released cyanide are rarely described in the literature, the effects of SNP itself should be evaluated in conjunction with the release of cyanide. 

Conversely, *S*-nitrosothiols such as *S*-nitroso-*N*-acetyl-penicillamine (SNAP) and *S*-nitrosoglutathione (GSNO) serve as physiologically close agents, as their NO release is often catalyzed by transition metals or light exposure, mimicking endogenous signaling pathways [47,90]. Moreover, naturally occurring *S*-nitrosothiol, *S*-nitrosoglutathione (GSNO), is regulated by endogenous enzymatic systems, e.g., GSNO reductase or cellular reductants (GSH, ascorbate, etc.) [47], which means that *S*-nitrosothiols’ NO liberation is tightly regulated. *S*-nitrosothiols are prone to photodegradation, although to a lesser extent than SNP, and GSNO half-life is encountered up to 7 h [91]. To prevent rapid decomposition of *S*-nitrosothiols, some controlled release systems were developed, such as chitosan-based nanoparticles, to provide a sustained NO supply during prolonged stress [105,106].

NONOates are not widely used in plant research as NO donors [79]. However, their structural diversity provides the researcher with a large set of molecules that might differ in their chemical properties. They exhibit predictable kinetics of NO release and a wide range of half-lives (from second to hours), working pHs, and temperature diapasons [94] and are not strongly influenced by biological molecules [80], implying that their practical use could be relatively broad depending on plant species, tissue, and type of stressor. These same properties make NONOates a “double-edged sword” in the hands of a researcher: their diversity and dependence on factors such as temperature and acidity of the environment require a large-scale screening of this class of compounds in plant systems and further careful selection of the most effective molecules [81]. In addition, plant NO research has historically relied more heavily on SNP and GSNO, making NONOates less established as routine tools [77,78].

Despite the NO donor nature, it is worth noting that many details about the dependence of nitric oxide release on physical and chemical factors have been studied *in vitro*. This idea is clearly illustrated by the work of Ederli and colleagues [78], who investigated NO release from NOS, SNP, and GSNO in solution and used *Nicotiana tabacum* as a model. The study demonstrates that NO donors differ markedly in NO release amounts: SNP releases the highest NO amounts in solution, whereas in leaves NOS is the most effective (ten times more NO than SNP). GSNO appears to be the least effective *in planta* in this particular model, failing to induce alternative oxidase (AOX1a) expression, hydrogen peroxide upbuilding, or cell death, in contrast to SNP and NOS. Although SNP and NOS generate different amounts of nitric oxide and SNP releases cyanide ion, the molecular effects they evoke seem to be common.

### 4.2. Delivery Modes of Nitric Oxide in Plants

**Figure 1:**
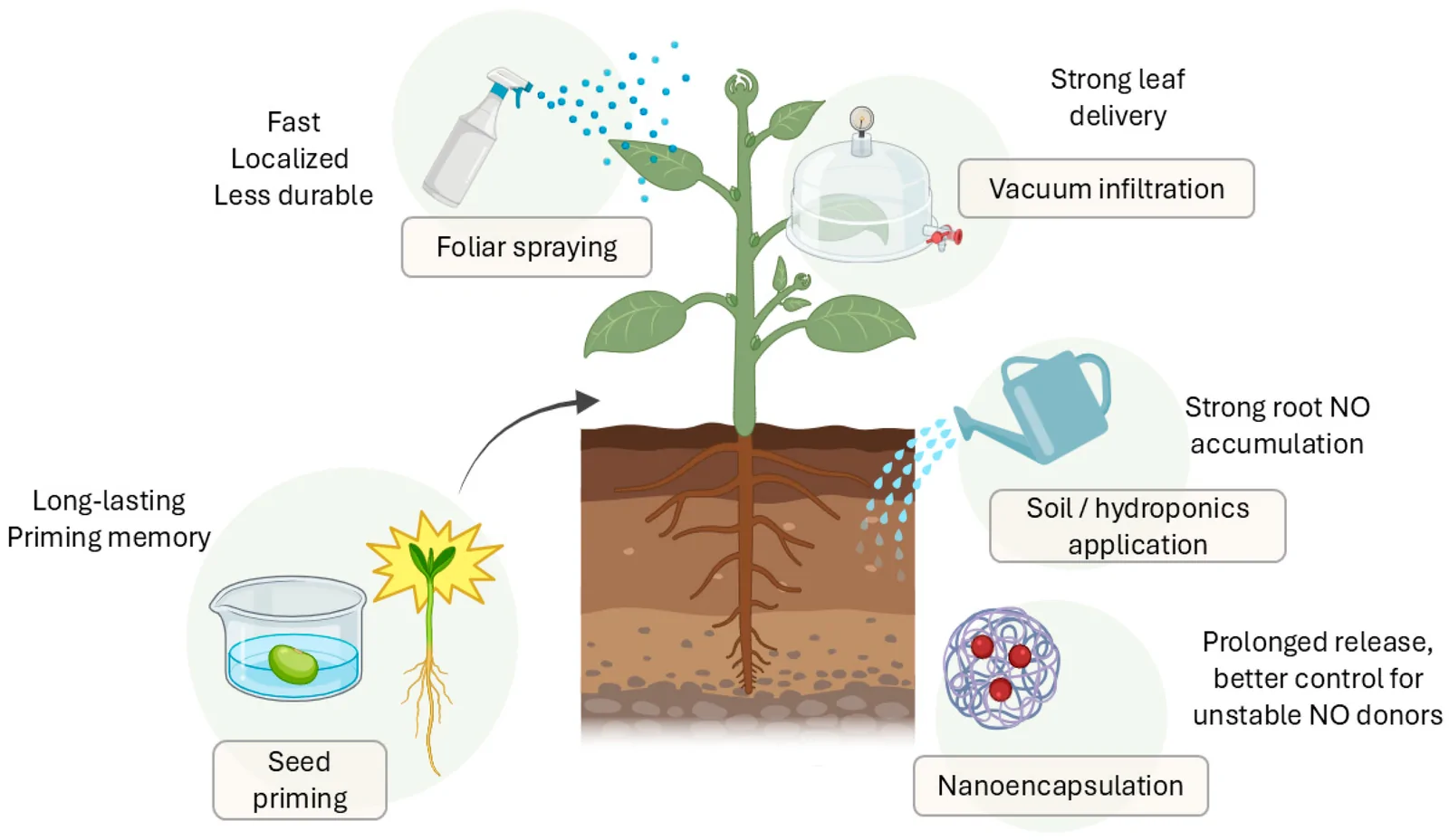
Graphical representation of the most common delivery modes of NO donors in plants. NO release kinetics, persistence of signal, ability, and strength to induce stress tolerance is highly dependent on the application method. Created in BioRender. Gurina, A. (2026) https://BioRender.com/3d0v2wv.

The way a NO donor is applied on plants highly influences the output: it determines the amount of NO accumulating in tissue, persistence of the signal, and even if the protective effects occur at all. The tests of the application method for the particular NO donor and particular plant species matter as they could vary greatly from case to case. A generalized scheme of the most used delivery methods is presented in Figure 1.

The research of Filippou et al. [107] on *Medicago truncatula* and SNP as a NO donor demonstrated that vacuum infiltration was the most effective in leaf tissue. On the other hand, hydroponic application produced the highest amount of NO in roots, with nitrate reductase activity correlating with NO levels in the tissue. This method of application of NO is not commonly used in plant research; however, this comparative study showed that vacuum infiltration is the most effective for leaves.

Seed priming also gained special attention: the results of the work by Habib et al. on wheat showed that seed soaking in SNP solution improved yield, photosynthetic pigments content, osmolyte accumulation, and antioxidant defense [108]. Another study on wheat confirmed that seed priming with NO donors evokes “priming memory”: in the study of Hameed et al., this type of treatment resulted in greater yield, plant biomass, and seed weight [109]. In a biotic stress example, seed priming with SNP for 4 hours alleviated nematode stress-reducing gall formation, stress markers, and improving antioxidant defense with phenolic compound contents in tomato seedlings [110]. This example designates that seed pre-treatment could be effective both for biotic and abiotic stresses. 

Leaf spraying has fewer durable effects, although it provides a fast-acting rescue. In lettuce under salt stress, spraying the leaves with SNP even just twice (after stress application) induced ascorbic acid, total phenols, and antioxidant enzymes contents [111]. Conversely, in salt-stressed strawberry, SNP spraying for one week before stress onset resulted in greater activity of catalase and ascorbate peroxidase and fruit yield compared with simultaneously or after stress application [112]. In the salt stress cotton model, three ways of SNP supplementation were tested: direct soil application, foliar spraying, or encapsulated slow-release methods [113]. Authors found out that foliar spray of 0.09 mmol/L SNP was best suited for growth and enzyme induction, whereas particle-bound SNP outperformed two other methods by the ability to induce accumulation of greater amounts of osmoprotectants. That is, it could be suggested that, at least in several cases, prophylactic spraying could be more effective than application during a stressful period. Nevertheless, spraying the leaves after exposure to a stressor can still improve the condition of the plants, making it a good choice in the case of unexpected severe conditions or for targeted treatment of the leaves only if other organs are not affected.

For GSNO specifically, encapsulation with nanoparticles substantially changes efficiency. In *Brassica napus*, root application of chitosan-GSNO particles resulted in greater amounts and prolonged NO release in vitro than free GSNO. It also induced accumulation of NO and efficiency in the induction of *S*-nitrosothiol signaling [114]. Expanding this knowledge to stress models, Souza and coworkers revealed that seed priming of wheat with encapsulated (chitosan/tripolyphosphate nanoparticles) GSNO and *S*-nitroso-mercaptosuccinic acid (MSNO) led to improved germination rates, chlorophyll content, and root morphology characteristics. The beneficial effect was also observed in the greenhouse [115]. Thus, the use of GSNO is currently shifting towards the development of NO-releasing nanomaterials, which is a more controllable and less degradable form. However, SNP is still the NO-releasing compound of choice independent of application method due to well-studied effects. Nevertheless, some side effects challenge researchers to develop new forms of NO donors and methods for their delivery in plant tissue.

Therefore, the effects of NO application should be interpreted as the combined result of donor chemical features and the delivery route. Even being “true” NO donors (i.e., releasing only nitric oxide), they can produce distinct metabolic and protein responses [87]. Omics technologies provide very valuable information in this case, as they allow us to distinguish between the effects of different methods of applying the substance and interpret their comparative effectiveness [116]. Combined multiomics studies also show that donors of different chemical natures are capable of inducing differential accumulation of distinct subsets of proteins and metabolites, thereby confirming that the chemical properties of individual compounds can significantly influence downstream pathways [87]. Therefore, omics technologies allow us to detect pathways associated with distinct formulations of the same compound. However, to distinguish these contributions, adequate comparison groups should be used (for example, only a donor, only a carrier, or the full composition) and the obtained molecular signatures should be interpreted together with quantitative data on the release of nitric oxide or its level in tissues, as well as with key physiological indicators [117]. This approach offers a possibility to identify whether the pathways are associated with the donor molecule, the carrier/delivery mode, or the full composition, although this strategy has not yet been systematically applied in studies of nitric oxide donors in plant research. 

### 4.3. Recent Advances in Novel NO Donors’ Design

Although well-known NO donors have already proven to be effective in treating plant stress, the design of completely new representatives continues to evolve. The creation of new representatives and even classes of NO donors aim to improve the predictability of nitric oxide release, regulate bioavailability by creating variously derivatized substances, and promote more predictable chemistry in general. They are classified into three groups: molecules with a dual donor function that are not nitric oxide releasers, endogenous NO metabolites that function as donors or transport vehicles, and donors that require an external trigger (Table 2).

One of the examples of dual donors are the NOSH molecules, which can release both NO and H_2_S. NOSH-aspirin is a special representative as it also delivers aspirin (acetylsalicylic acid)—an additional bioactive compound, especially regarding salicylates’ protective role in plants [122]. In *Medicago sativa*, foliar spraying before drought improved subsequent acclimation and recovery after stress, reflected in better physiological characteristics and altered transcriptional control of defense pathways [118]. Singh and colleagues reported the synthesis of *N*-nitrosomelatonin (NOMela) [119]; when *Arabidopsis* seedlings were incubated in 250 μmol/L NOMela solution, the compound was readily uptaken by primary roots and efficiently transported in leaves. NOMela was a more productive NO transporter than GSNO *in planta* and released more than 50% greater NO than GSNO *in solution*. Although the research on NOMela efficiency in plant stress models is limited, the usage of this molecule is still promising based on transporting and NO-release potency. 

Nitro-fatty acids are a good example of NO-derived metabolites that serve as physiological NO donors: nitro-linolenic acid (NO_2_-Ln) was shown to release NO and modulate GSNO synthesis directly in *Arabidopsis* using mass spectrometry-based approaches [120]. Pre-treatment of roots with NO_2_-Ln induced HSPs transcripts in *Arabidopsis*, supporting the fact that exogenous application could also regulate stress responses [123]. 

A conceptually different approach in NO donor design is the creation of representatives with controlled release of NO in space and time (e.g., by light beams or another external trigger). For instance, by applying photoactivatable the nitric oxide (NO) donor *N*-nitroso-naphthalene derivative P1G3 to *Arabidopsis* roots, authors used light (varying in duration and intensity) to control nitric oxide release and were able to track this event in tissue due to the formation of fluorescent product [121]. That molecule induced guard-cell Ca^2+^ accumulation and stomatal closure, pointing to drought protection by spatiotemporally controlled NO delivery. This is a completely new approach to regulate NO release, which has not yet been widely studied in the literature. Tert-butyl *S*-nitrosothiol (tBuSNO) and tert-dodecane *S*-nitrosothiol (tDodSNO) are other photoactivatable drugs with improved NO release kinetics (compared with other conventional nitrosothiols) [124]. While GSNO or SNAP decompose rapidly and are uncontrollable, tBuSNO and tDodSNO could be targeted to particular tissue or organ and then precisely activated by a beam of light [125]. However, the studies on plants employing these substances are lacking. 

While advancing NO delivery, the novel donors bring new opportunities: the generation of controlled NO release (by nanoencapsulation, structural features, or external stimuli) allows for more precise mapping of molecular cascades and the main NO-modified targets. Single-omics and multiomics approaches, due to their high-throughput nature, are the preferred methods for resolving complex NO signaling networks in plant stress.

Despite the fact that the creation of new NO donors is an ever-growing field, at the moment, they are either incompletely characterized or only *in vitro*. The compounds described in this section has already shown their beneficial effects in plants; however, statements about agricultural potential should be interpreted cautiously. For these compounds, systematic data on stress alleviation on different levels (transcripts, proteins, PTMs, and metabolites) is still limited. Furthermore, most of them were tested only on model organisms such as *Arabidopsis*. Filling these gaps will require standard quantification of NO in plant tissues, studies on dose dependence across species (most importantly, food crops), and integration of physiological data with single- or multiomics to discriminate true NO effects from the effects of the whole compound.

## 5. Multiomics in the Deciphering of NO Donor Mechanisms

### 5.1. Perspectives of the Application of Multiomics in Studying the Effect of Nitric Oxide (NO) Donors on Plants

Omics technologies comprise a diverse set of methodological platforms originally developed to investigate individual levels of genetic information flow [126]. Their rapid expansion became possible in the post-genomic era, when the genomes of major model organisms had been deciphered, providing the foundation for transcriptomics and proteomics [127]. Later, additional omics fields emerged, including metabolomics and phenomics, and the list continues to grow as new analytical platforms are developed. Omics technologies are now routine tools for studying plant responses to diverse environmental and biological factors because they enable the simultaneous identification and analysis of thousands of molecules. This makes it possible to build a biological interpretation based on changes in transcript abundance, protein accumulation, and metabolite levels, thereby characterizing the activation or repression of specific metabolic pathways [128]. Compared with the analysis of individual enzymes or single genes, omics approaches are far more productive and integrative [129]. Analysis of post-translational modifications of proteins and, at the transcriptome level, alternative splicing, alternative polyadenylation, RNA editing, and non-coding RNA-mediated regulation, provides an additional layer of insight into response regulation that cannot be explained by expression changes alone. 

From this perspective, integrating multiple omics disciplines allows for a more holistic understanding of the biological phenomenon being studied. Transcript expression does not necessarily mean obligatory translation into protein, while protein accumulation data can be significantly complemented by differential metabolite accumulation data. On the other hand, as discussed in previous paragraphs, NO acts both on transcriptional and PTM levels. Thus, multiomics is a valuable tool in understanding the deep molecular mechanisms underlying NO signaling and protective effects of NO donors on unstudied species of plants, as well as the detailed action of novel NO donors too.

### 5.2. Transcriptomics: Reprogramming the Stress Transcriptome

Transcriptome studies on various plant species show that nitric oxide donors, particularly SNP, induce large-scale reprogramming of both coding and non-coding RNAs to enhance stress resistance. Despite differences in species, stressors, and experimental design, trascriptomics analysis revealed some conserved modules, centered on antioxidant defense, metal homeostasis, hormone signaling, and microRNA-mediated regulation. These results suggest that nitric oxide-mediated transcriptional activation involves conserved pathways, including antioxidant defense, metal homeostasis, hormonal signaling, and microRNA-regulated networks. Moreover, transcriptional factors are controlled by *S*-nitrosylation (as was described in the Section 3.2), thereby influencing TF’s transport and ability to bind their target sequences [130,131].

Analysis of the transcriptome of alfalfa regulated by SNP in osmotic stress revealed massive transcriptional reprogramming and highlighted molecular markers of redox homeostasis and stress signaling, such as phytochrome-interacting factor 3 (PIF3), GSTs, SOD2, GPX, and respiratory burst oxidase homolog (RBOH) as the most SNP drought-responsive by expression levels [132]. In a rice study addressing arsenic stress, transcripts corresponding to various metal transporters were upregulated upon SNP application in roots [133]. These included NOD26-like intrinsic protein (NIP), natural resistance-associated macrophage protein (NRAMP), and ATP-binding cassette (ABC). Stress-related genes such as cytochrome P450 (CytP450), GSTs, glutaredoxin (GRXs), and TFs were also upregulated, along with hormone, signaling, and detoxification pathways. By these examples, it is clear that donor-emerging NO provides massive transcriptomic reprogramming and activates conserved transcriptional networks across species. 

More recent studies consider not only the expression of protein-coding mRNAs, but also non-coding RNAs with various functions. Analysis of alfalfa’s miRNAs repertoire demonstrated that SNP and drought treatment was accompanied not only by the expression of previously known transcripts but also by some new ones. For that set of RNAs, target genes and their functions were identified [134]. The role of miRNA was addressed in detail in other studies using alfalfa: NO (derived from SNP) reduced Msa-miR166 expression under short-term drought, targeting homeodomain-leucine zipper III (HD ZIP-III), FRIGIDA (FRI), and CoA ligase genes [135], while NO-regulated Ms-miR398a-3p was found to cleave Cu/Zn SOD transcript, thus linking redox signaling and drought adaptation responses [136]. In citrus, RNA-seq analysis revealed that SNP priming before drought activated the expression of genes related to photosynthesis, antioxidant capacity, energy reallocation, and external barriers reinforcement, as well as components of synthesis and signaling of auxins [137]. 

### 5.3. Proteomics and Post-Translational Modification Profiling

While transcriptomics provides large data on differential gene expression and the presence of some regulatory RNAs, this information is not connected directly to functional phenotype [138]. Proteomics is precisely the field that studies the next level of genetic information implementation and the proteins that carry out the main cellular reactions—metabolic or regulatory—bringing science closer to understanding the underlying mechanisms.

The application of different NO donors in the research of plant stress by means of proteomics allowed for the reveal of massive reprogramming of plant metabolism upon adverse environments. A donor comparison study using SNP, diethylenetriamine (DETA), and GSNO was carried out in chickpea, and proteomic analysis revealed 172 and 76 down- and upregulated proteins. The authors highlight that a majority of them were chloroplastic, implying the importance of local NO signaling in these organelles [139]. This observation could be complemented by various studies on plants in stress conditions, where the addition of a NO donor was accompanied by accumulation of photosynthetic proteins [140,141,142]. Apparently, the NO-regulated proteome is not limited to photosynthetic function: in salinity-stressed barley, the donor induced various proteins responsible for photosynthesis, stress defense, and degradation of damaged proteins [143]; in a study on banana roots, SNP-associated protein shifts were associated primarily with carbohydrate and energy metabolism [144]. The main caveat is that SNP is not a clear NO donor, which was discussed in the proteomics work by Keisham and coworkers, which demonstrated salinity- and SNP-regulated proteins and segregated the effects of NO, cyanide, and iron released by SNP [145]. 

The development of nitroso-proteomics within the proteomics approach has allowed us to understand how the NO signal can be transmitted through protein targets. Thus, currently, proteomics is moving from describing the processes involving NO-regulatory proteins to studying the role of *S*-nitrosylation and its impact on protein activity. A *Gossypium hirsutum* example provided an additional possible role of nitric oxide in stomata closure independently from ABA: under drought stress and utilizing SNP, the authors found that plasma membrane H^+^-ATPase isoform GhHA2 is *S*-nitrosylated and thereby stabilized from proteasomal degradation [146]. Another group of authors supposes that NO controls even stomata development and stress responses. This is because mitogen-activated kinase 6 (MPK6) could be nitrosylated, which inhibits the kinase activity. The mitogen-activated protein kinase signaling cascade MPK3/MPK6 pathway is the main regulatory network in stomata developmental programs [147].

Nitric oxide directly regulates hormone signaling networks: for example, a DELLA protein family member (key negative regulators of the gibberellin signaling pathway) was found to be nitrosylated both in GSNO/SNP treatments and endogenously (employing *gsnor1-3* and *nox1* mutants). This prevents its proteasomal degradation and promotes salt stress tolerance [148]. *S*-nitrosylation also acts on a deeper, gene expression-regulating level and epigenetic control. A study dedicated to redox regulation of SRG1 (plant immunity-related transcriptional factor) on *Arabidopsis* showed that SRG1 could be NO-modified (endogenously or with GSNO application), which serves as a negative feedback regulation of response to a pathogen [71]. *Arabidopsis* histone deacetylase HDA19 *S*-nitrosylation can occur under stress conditions endogenously or induced by GSNO, and this modification promotes deacetylation of target genes to improve tolerance to oxidative stress [149].

Another study focused on plasma membranes under cadmium stress: a quantitative proteomics approach using isobaric tag for relative and absolute quantitation (iTRAQ) demonstrated differential expression of various transporters, ATPases, kinases, metabolic enzymes, phosphatases, and phospholipases, where abundance of phospholipase D rose substantially under Cd and Cd plus NO donor treatments [150]. Further investigations of subproteomes of particular cellular compartments would clarify a special role of them in sophisticated NO signaling networks.

### 5.4. Metabolomics: Metabolic Reprogramming Under NO Priming

Metabolomics profiling significantly complements other omics studies, providing more specific information on the metabolic adjustment emanating from the altered enzyme activity or protein expression levels. However, this is also independent information, for example, about the accumulation of osmolytes, antioxidants, and other stress-protective substances, as well as about changes in the content of valuable nutrients (amino acids, sugars) under the influence of NO, which is especially relevant for the fruits of food crops.

Among the metabolic changes observed after the administration of nitric oxide donors, one of the most stable is the accumulation of osmolytes [151,152]. Rehaman et al. captured augmentation of some antioxidative compounds (tocopherols, sterols, etc.) and membrane-protecting compounds (triterpenoids α-amyrin, and friedelan-3-one) besides proline accumulation in common bean leaves after SNP application [153]. SNP application to *Fatsia japonica* significantly rescued salt-stressed phenotype, improving sugar, amino, and organic acids levels [154]. The white clover study is particularly informative because, although SNP has some common metabolic effects with gamma-aminobutyric acid (GABA) and mannose (such as higher levels of myo-inositol and mannose), it also caused a specific increase in citric acid associated with the tricarboxylic acid cycle (TCA) cycle, indicating that NO-based priming creates a distinct metabolic signature that cannot be simply replaced by other priming compounds [155]. 

NO donors also frequently induce accumulation of non-enzymatic antioxidants. The pepper study provides a stress-specific illustration: under high temperature, scavenging NO with carboxy-PTIO [156] intensified oxidative damage and disrupted enzyme activities, whereas supplying NO via SNP restored growth by strengthening antioxidant defenses and normalizing amino acid metabolites such as kynurenine, *N*-acetyl-*L*-tyrosine, *L*-methionine, urea, and creatine, highlighting that protection relies on rebalancing nitrogen and amino acid flux rather than simply boosting classical antioxidant enzymes [157].

In general, standalone metabolomics studies on NO signaling role are more scarce than proteomics or transcriptomics ones. However, a substantial part of them is included in integrated multiomics research, providing supporting data as a missing piece of the puzzle and reflecting almost phenotypic, but still molecular-level results [117].

### 5.5. Integrated Multiomics Analysis

Although the story of nitric oxide in plant stress research started relatively long ago, multiomics studies are still more scarce than single-omics or omics–physiology/biochemistry ones. Most of the existing multiomics papers studying the role of NO in plant stress alleviation rely on transcriptomics and metabolomics integration, which could be explained by some key reasons. First, transcriptomics and metabolomics data analysis became more efficient and cost-effective due to the development of high-throughput technologies [158]. Conversely, proteomics workflow is usually higher in cost [159]. Second, the transcriptomics–metabolomics bundle could be more reliable when the biological question is “How transcriptional reprogramming induced by NO is reflected in metabolic adjustment”; transcriptional changes reflect upstream regulation, while metabolites are closer to the phenotype [158]. However, proteomics data could be useful in this context too, as it directly measures protein abundance (not just presence of transcript) [160] and captures PTMs, which add a unique level in the regulation of protein activity [161] and are especially valuable in the assessment of NO function.

Exogenous NO donors reinforce secondary metabolite-based defense programs (waxes, stilbenes, phenylpropanoids, flavonoids) through coordinated transcriptional and metabolic reprogramming. Combined gas chromatography–mass spectrometry (GC–MS) and transcriptomics analyses identified the major role of cuticular wax formation in *Lagenaria siceraria* induced by SNP under drought [162]. Similarly, integrated metabolomics and transcriptomics analysis revealed that exogenous NO application could confer tolerance of grape berry to *Botrytis cinerea*. This was explained by increased expression of genes in the antioxidant-associated stilbene synthase pathway (including stilbene and chalcone synthase) and related metabolites [163]. The importance of secondary metabolites regulated by exogenous NO in protecting plants from stress is further emphasized by a study on *Sorbus pohuashanensis*. Here, an integrated transcriptome–metabolome approach showed NO-dependent upregulation of phenylpropanoid and flavonoid biosynthetic genes. These included phenylalanine ammonia-lyase (PAL), cinnamate 4-hydroxylase (C4H), ferulate 5-hydroxylase (F5H), 4-coumarate-CoA ligase (4CL), and chalcone synthase (CHS). Concrete shifts in phenylpropanoid/flavonoid and related metabolites were also observed [164].

Proteomics studies combined with other omics are relatively scarce in this field. However, they demonstrate that the addition of a proteomics or nitrosoproteomics level clarifies connection of transcriptional reprogramming and protein-level adjustments, which with *S*-nitrosylation assays directly point out the proteins regulated by this PTM.

In a tomato–salt stress study, GSNO was used as the NO donor, and *S*-nitrosoproteomics revealed nitrosylation of MAPK proteins, which were differentially up- or downregulated at the transcriptional level. Enzyme assays plus metabolite measurements identified activation of glycolysis and the TCA cycle as key carbohydrate metabolism responses conferring salt tolerance [63]. In tomato seedlings under low-nitrogen stress, transcriptome analysis allowed for the identification of 657 differentially expressed transcripts enriched in photosynthesis, secondary metabolism, MAPK, and nitrogen metabolism pathways, which was complemented by nitrosoproteomics analysis. The latter demonstrated *S*-nitrosylation of the MAPK signaling pathway and carbohydrate metabolism components enrichment under low nitrogen conditions [165]. These studies are valuable examples of productive usage of proteomics (namely, nitrosoproteomics) and transcriptomics in practice. However, a clear research gap could be seen as there is no good representation of such multi-layered investigations in the literature. Integrated datasets can connect donor-induced gene reprogramming to downstream metabolic and physiological outcomes. They are even more informative when proteomic or nitrosoproteomic layers are added. This is because NO frequently acts through post-translational regulation, especially *S*-nitrosylation, which cannot be inferred reliably from transcript levels alone.

One of the possible obstacles to studying NO signaling using multi-omics technologies is the complexity of integrating and interpreting the obtained data [166]. The most direct path is biochemical pathways. However, certain difficulties arise here, related to post-translational modifications and regulatory issues (including microRNAs). Metabolism is regulated by multiple factors: other genes, microRNAs, and signaling pathways. Therefore, a change in the level of one metabolite may be the result of the operation of an entire network, not just a single gene [167]. The more informative approach is correlation analysis. In research, a matrix is often constructed, where the rows represent genes (or proteins), the columns represent metabolites, and the cells contain correlation coefficients between the level of gene expression (or protein quantity) and the concentration of a metabolite under different conditions [168]. Here, it is important to eliminate false correlations: for example, the relationship may be due to a common external factor (stress, diet) rather than a direct cause-and-effect relationship [169]. Analysis of functional groups can be performed if genes can be grouped by biological processes, molecular functions, or pathways (e.g., from the Kyoto Encyclopedia of Genes and Genomes, KEGG) [170]. The connection is first identified using bioinformatics and then verified experimentally; for example, computationally predicted regulatory interactions were further tested with gain- and loss-of-function mutants [171]. For example, qRT-PCR is used to confirm the mRNA level for a candidate gene, and immunoblotting or other methods are used to determine the protein level [167,168]. In real-world studies, networks are often constructed where the nodes represent genes, proteins, or metabolites, and the connections represent statistically significant correlations or regulatory pathways. Specialized tools (for example, Cytoscape) are used to visualize and analyze such networks [172]. There are also specialized packages for R (such as mixOmics or ShinyGAM) that help integrate data from different omics [166]. mixOmics includes methods such as DIABLO, which selects features that capture shared information across omics datasets while distinguishing between phenotypic groups [173]. The scale of the data is also a problem. In real studies, the lists of differentially expressed genes, proteins, and metabolites are often long. Careful preprocessing, appropriate correction for multiple comparisons, and validation in independent samples or experiments are therefore essential when drawing conclusions about NO-responsive pathways [168].

## 6. Conclusions

Currently, the role of nitric oxide in enhancing plants’ stress resistance is being actively studied from various perspectives. However, research shows that the initial focus on well-known donors such as SNP or GSNO is beginning to shift, and new research is being directed towards finding new formulas or creating other ways to deliver nitric oxide to tissues (such as encapsulation). Thus, the study of NO targets and the selection of completely new compounds are relatively parallel. Nitric oxide contributes to the modulation of plant responses to stressors, but is applied exogenously, and its effects strongly depend on chemical properties, dose, timing, and delivery mode. For example, nanoencapsulation of commonly used GSNO appears to be more promising as this formulation alters release kinetics and improves stability relative to the free-donor form. 

Different delivery routes could shape distinct molecular responses in plants, and their suitability could vary depending on stress nature, plant species, and the particular compound. Vacuum infiltration and hydroponic delivery are optimal for achieving high and organ-specific NO levels, but they are more technically complicated. Seed priming shapes durable and organism-level priming memory, whereas foliar spraying provides rapid rescue. At the organellar level, chloroplasts, mitochondria, and peroxisomes are plausible sites for further targeted investigation of NO hubs because they affect photosynthesis, cellular respiration, and redox pathways in these compartments.

The most highly productive and reliable methods are omics approaches, which allow us to analyze the characteristics of plant stress responses at the molecular level. Despite the well-established role of single-omics and multiomics technologies in studying plant responses to stress, the study of the role of nitric oxide using these methods is still in its early stages. Future research will significantly contribute to this picture by establishing a link between specific sources of nitric oxide and high-resolution molecular maps. Future studies should address the key unresolved questions: which molecular alterations are induced by NO itself rather than the donor or carrier themselves; which NO-responsive pathways are principal for stress tolerance; and whether phytoeffective properties be preserved in the field. Addressing these questions to clarify tissue- and organelle-scale features of NO signaling and experimental validation of multiomics predictions could help develop new forms of NO-donating agents (or improve existing forms) that are more precise, reproducible, and appropriate to specific crop–stress combinations. This will allow nitric oxide to be transformed from a broad-spectrum “protective agent” into a finely tuned stress-resistance regulator that can be rationally used in agriculture. 

## Figures and Tables

**Table 1:** Comparison of the major groups of nitric oxide donors applied in plant stress research.

Comparison Axis	Sodium Nitroprusside (SNP)	*S*-Nitrosoglutathione (GSNO)	Diazeniumdiolates (NONOates)	References
Stress Effects	Alleviates multiple abiotic stresses at optimal doses; high doses cause nitrosative stress, growth inhibition, lipid peroxidation, altered antioxidant activity, and cell death	Roles in redox regulation, *S*-nitrosylation and stress acclimation; often used to probe NO-dependent signaling rather than protection	Potential for precisely timed stress mitigation, but experimentally underexplored, with outcomes highly context- and derivative-dependent	[47,77,78,79,80,81,82,83,84,85,86,87,88,89,90]
NO delivery behavior	Rapid NO release in solution; relatively long half-life (up to ~12 h) and pronounced photosensitivity	Catalyzed by light or transition metals; enzymatically regulated by GSNOR; moderately photolabile	Highly predictable and tunable release kinetics (seconds to hours), defined pH and temperature windows; largely unaffected by biological molecules	[47,78,80,86,90,91,92,93,94]
Are the effects truly “NO-like”	Partially; cyanide co-release means that some physiological effects (e.g. photosynthetic inhibition, seed dormancy break) may reflect cyanide as well as NO	More closely “NO-like” and physiologically relevant	Conceptually very NO-specific (clean NO release, minimal side products), but plant data are insufficient	[47,77,78,79,80,81,90,95,96]
Efficiency costs	Cheap and easy to use; requires careful concentration control and consideration of non-NO side effects	More expensive and less stable than SNP; photodegradation and reliance on metal catalysts/reductants demand controlled handling	Chemically versatile but demanding: many derivatives, each with specific stability and release profiles, require extensive pre-screening and optimization; not yet supported by broad plant practice	[47,77,78,79,81,83,84,89,90,91,94,95,97,98]

**Table 2:** Emerging classes of nitric oxide donors in plant research.

Class of Novel NO Donor	Representative Molecules	Key Features	Reported Stress-Related Effect(s) in Plants	References
Dual donor molecules (NO + another bioactive gas/compound)	NOSH-aspirin (NO + H_2_S + acetylsalicylic acid)	Simultaneous release of NO and H_2_S; acetylsalicylic acid as additional bioactive compound linked to salicylate signaling	Foliar spraying before drought improved physiological performance and recovery after stress in Medicago sativa; altered transcriptional control of defense pathways	[118]
Dual donor molecules (NO + another bioactive gas/compound)	*N*-nitrosomelatonin (NOMela)	Melatonin-derived NO donor/transporter; higher NO release potency than GSNO in solution and efficient root-to-shoot transport	In Arabidopsis, readily taken up by primary roots and transported to leaves; promising as NO donor for stress studies, though direct stress alleviation data remain limited	[119]
Endogenous NO-derived metabolites acting as physiological NO donors/transporters	Nitro-linolenic acid (NO_2_-Ln)	Plant-derived nitro-fatty acid that can release NO and modulate GSNO synthesis; identified by mass-spectrometry-based approaches	In Arabidopsis, pre-treatment of roots induced heat-shock protein (HSP) transcripts, supporting the capacity to regulate stress-responsive gene expression	[120]
Externally triggered (photo-controlled, spatiotemporal) NO donors	*N*-nitroso-naphthalene derivative P1G3	Light-controlled NO release (duration and intensity of illumination as external trigger); formation of fluorescent product enables in-tissue tracking	In Arabidopsis, induced guard-cell Ca^2+^ accumulation and stomatal closure, indicating the potential for drought protection via spatiotemporally controlled NO delivery	[121]

GSNO—*S*-nitrosoglutathione; H_2_S—hydrogen sulfide; NO—nitric oxide; NO_2_-Ln—nitro-linolenic acid; NOMela—N-nitrosomelatonin; NOSH-aspirin—nitric oxide and hydrogen sulfide-releasing aspirin.

## Data Availability

No new data were created or analyzed in this study. Data sharing is not applicable to this article.

## Abbreviations

The following abbreviations are used in this manuscript:

ABA	abscisic acid
ABC	ATP-binding cassette transporter
ACS	1-aminocyclopropane-1-carboxylate synthase
APX	ascorbate peroxidase
ATP	adenosine triphosphate
CAT	catalase
CHS	chalcone synthase
CO	CONSTANS (gene, abbreviated)
DAF	diaminofluorescein
DELLA	DELLA proteins
DETA	diethylenetriamine NONOate
DHAR	dehydroascorbate reductase
EPR	electron paramagnetic resonance
ERFVII	ethylene response factor VII
ESR	electron spin resonance
ETC	electron transport chain
FRI	FRIGIDA (gene, abbreviated)
FRIGIDA	FRIGIDA gene (regulator of flowering time)
GABA	gamma-aminobutyric acid
GC	gas chromatography
GI	GIGANTEA (gene, abbreviated)
GPX	glutathione peroxidase
GR	glutathione reductase
GSH	reduced glutathione
GSNO	*S*-nitrosoglutathione
GSNOR	*S*-nitrosoglutathione reductase
GST	glutathione *S*-transferase
HD	homeodomain
HSP	heat shock proteins
KEGG	Kyoto Encyclopedia of Genes and Genomes
LEA	late embryogenesis abundant proteins
MAP	mitogen-activated protein
MAPK	mitogen-activated protein kinase
MDHAR	monodehydroascorbate reductase
MS	mass spectrometry
MSNO	*S*-nitrosomercaptosuccinic acid
NADH	nicotinamide adenine dinucleotide (reduced form)
NADPH	nicotinamide adenine dinucleotide phosphate (reduced form)
NIA	nitrate reductase gene (in plants)
NIN	nodule inception transcription factor
NIP	NOD26-like intrinsic protein
NIR	nitrite reductase
NO	nitric oxide
NOS	nitric oxide synthase
NOSH	*S*-nitrosothiol
NR	nitrate reductase
NRAMP	natural resistance-associated macrophage protein (metal transporter)
PAL	phenylalanine ammonia-lyase
PEG	polyethylene glycol
POD	peroxidase
PTIO	2-phenyl-4,4,5,5-tetramethylimidazoline-1-oxyl-3-oxide
PTM	post-translational modifications
RBOH	respiratory burst oxidase homolog (NADPH oxidase)
RGB	Red–green–blue
RNA	ribonucleic acid
RNS	reactive nitrogen species
ROS	reactive oxygen species
SNAP	*S*-nitroso-*N*-acetylpenicillamine
SNP	sodium nitroprusside (nitric oxide donor)
SOD	superoxide dismutase
SP	signal peptide
TCA	tricarboxylic acid cycle (Krebs cycle)
UV	ultraviolet
ZIP	leucine zipper
